# Supplementary material for: A Clinicopathological Review of 203 Cases of Atypical Polypoid Adenomyoma of the Uterus
Source: J Clin Med. 2023 Feb 14;12(4):1511. doi: 10.3390/jcm12041511 (PMC9966326; doi:10.3390/jcm12041511)
Supplement: Supplementary file 1 [file jcm-12-01511-s001.zip › jcm-2167504-supplementary.pdf]

Table S1 Immunohistochemical markers of APA in our study

Immunohistochemical staining %(N)					
Glandular	ER (+)	94.8%（92/97）	Stromal	CD10 (-)	89.5%（34/38）
	PR (+)	94.8%（92/97）		p16 (+)	86.9%（53/61）
	Ki-67＜30% (+)	51.5%（51/99）		h-caldesmon (-)	66.7%（2/3）
	Mutation-type p53	59.5%（47/79）		Desmin (+)	75.0%（3/4）
	PTEN (+)	18.8%（3/16）		Vimentin (+)	88.9%（16/18）
	CDX2 (+)	50.0% (1/2)			
	MLH1 (+)	96.4%（27/28）			
	MSH2 (+)	96.4%（27/28）			
	MSH6 (+)	96.4%（27/28）			
	PMS2 (+)	96.4%（27/28）			

Supplementary figure and figure legends

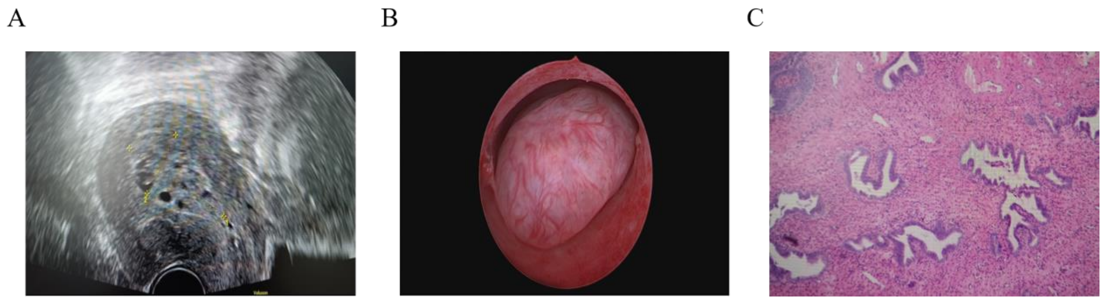

Figure S1 Representative image of APA in our study.

(A) Representative ultrasound image, (B) Representative hysteroscopical image, (C) Representative microscopic appearance (H&E stain).
